# Prognostic factors related to ambulation deterioration after 1-year of geriatric hip fracture in a Chinese population

**DOI:** 10.1038/s41598-021-94199-0

**Published:** 2021-07-19

**Authors:** Ronald Man Yeung Wong, Jianghui Qin, Wai Wang Chau, Ning Tang, Chi Yin Tso, Hiu Wun Wong, Simon Kwoon-Ho Chow, Kwok-Sui Leung, Wing-Hoi Cheung

**Affiliations:** 1 Department of Orthopaedics and Traumatology, The Chinese University of Hong Kong, Sha Tin, Hong Kong SAR; 2 Department of Orthopaedics and Traumatology, Prince of Wales Hospital, Hospital Authority, Sha Tin, Hong Kong SAR

**Keywords:** Trauma, Fracture repair

## Abstract

The objective of this study was to investigate the prognostic factors predicting the ambulation recovery of fragility hip fracture patients. 2286 fragility hip fracture patients were collected from the Fragility Fracture Registry in Hong Kong. Predictive factors of ambulation deterioration including age, gender, pre-operation American Society of Anesthesiologists grade, pre-fracture mobility, delay to surgery, length of stay, fracture type, type of surgery, discharge destination and complications were identified. Patients with outdoor unassisted and outdoor with aids ambulatory function before fracture had 3- and 1.5-times increased risk of mobility deterioration, respectively (Odds Ratio (OR) = 2.556 and 1.480, 95% Confidence Interval (CI) 2.101–3.111 and 1.246–1.757, both p < 0.001). Patients living in old age homes had almost 1.4 times increased risk of deterioration when compared to those that lived in their homes (OR = 1.363, 95% CI 1.147–1.619, p < 0.001). The risk also increased for every 10 years of age (OR = 1.831, 95% CI 1.607–2.086, p < 0.001). Patients in the higher risk ASA group shows a decreased risk of ambulation deterioration compared to those in lower risk ASA group (OR = 0.831, 95% CI 0.698–0.988, p = 0.038). Patients who suffered from complications after surgery did not increased risk of mobility decline at 1-year post-surgery. Delayed surgery over 48 h, delayed discharge (> 14 days), early discharge (less than 6 days), and length of stay also did not increased risk of mobility decline. Male patients performed worse in terms of their mobility function after surgery compared to female patients (OR = 1.195, 95% CI 1.070–1.335, p = 0.002). This study identified that better premorbid good function, discharge to old age homes especially newly institutionalized patients, increased age, lower ASA score, and male patients, correlate with mobility deterioration at 1-year post-surgery. With the aging population and development of FLS, prompt identification of at-risk patients should be performed for prevention of deterioration.

## Introduction

Fragility hip fractures are one of the most common injuries which is significantly associated with high morbidity and mortality^1^. More importantly, the recent concept of imminent risk of fragility fractures after an initial one has highlighted the importance in identifying the prognostic factors of deterioration^1,2^. In fact, approximately 50% of secondary fractures occur during this period of 2 years after the primary fragility fracture. Studies have postulated the cause due to sarcopenia resulting from the lack of rehabilitation and prolonged bedrest after an initial fracture^3^. Previous studies have shown that up to a half of all patients could not regain their pre-fracture ambulatory status after surgery^4^ and lost independence^5^. This has resulted in a huge economic burden, particularly with the aging society^6,7^.

Recent publications have also highlighted that ambulatory status is essential for quality of life in patients^8^. More importantly, optimal post-fracture care can be achieved with multimodal interventions combining physiotherapy, nutrition and pharmacology^9^. These intensive in-patient rehabilitation can allow physical function recovery, preventing secondary falls and fractures^10^. Therefore, identifying the factors and patients at risk of mobility deterioration is of utmost importance and priority to reduce functional decline and institutionalization^10^. As of now, there is still a lack of evidence and need for further studies for the identification of factors of functional outcomes after hip surgery and their underlying mechanisms^11^.

Previous studies have revealed a variety of factors including age, pre-fracture ambulation status, cognitive status, co-morbidities and rehabilitation treatment after discharge, that could potentially affect functional recovery^12–18^. However, findings have been largely inconsistent due to different methodologies for assessment in current studies^16–18^. More importantly, there has been a lack of large sample studies with patients ranging at hundreds only^16,19,20^ and few have focused on the Chinese population^21^. In fact, it is predicted that the number of hip fractures in Asia will increase from 1,124,060 in 2018 to 2,563,488 in 2050, with a 2.28-fold increase, and the majority will be from China^22^. Although not all risk factors are modifiable, identification can allow the clinician to be aware of the functional recovery to properly counsel the patient and their family. Further studies are therefore warranted to identify these important risk factors for a holistic clinical care. To our knowledge, we present the first large scale study in a Chinese population, which will potentially address a significant number of future patients.

In this study, consecutively enrolled geriatric hip fracture patients were assessed for risk factors. A series of pre-fracture and peri-surgical factors, including age, gender, pre-operation ASA (American Society of Anesthesiologists) grade, pre-fracture mobility, delay of surgery > 48 h, fracture type, type of surgery, length of stay, discharge destination and complications were studied to identify the potential predictors of mobility deterioration after a one-year follow-up.

## Methods

A post-hoc analysis of a prospective cohort study was performed. Consecutive patients were collected from the Fragility Fracture Registry program of Hong Kong (FFR; https://www.ffr.hk) with age 65 years old or older. All patients had a fragility hip fracture and were admitted in 2012 from six acute public hospitals under the management of Hospital Authority in Hong Kong. Patients that had atypical or pathological fractures or died during the study period were excluded. Ambulatory status in the database was categorized into six grades: grade 1 (fully ambulatory); grade 2 (ambulation with one aid); grade 3 (ambulation with frame); grade 4 (indoor confined ambulation); grade 5 (standing only) and grade 6 (non-ambulatory), based on hierarchy of ambulatory status^23^.

Patients were separated into “mobility regained group” (ambulatory status remain the same or improved) and “mobility deteriorated groups” (ambulatory status deteriorated) by comparing the previous ambulatory status with one-year after fracture. Predictive factors of ambulation deterioration were identified from age, gender, pre-operation ASA (American Society of Anesthesiologists) grade, pre-fracture mobility, type of surgery (bipolar hemiarthroplasty cemented or cementless, unipolar hemiarthroplasty cemented or cementless, total hip replacement cemented or cementless, excisional arthroplasty, cannulated hip screws, compression hip screw, cephalomedullary device, and no operation as anesthetically unfit), delay of surgery > 48 h, length of stay at acute orthopedic ward (LOS), discharge destination, complications (urinary tract infection, chest infection, pressure sore, delirium, deep vein thrombosis, wound infection, anemia), and fracture type (neck of femur fracture, intertrochanteric fracture, subtrochanteric fracture).

For feasibility of clinical practice, pre-fracture mobility was divided into three groups according to ambulatory capacity^19^. Patients were categorized as the outdoor unassisted group (ambulatory grade 1), outdoor with aids group (ambulatory grade 2 and 3) and indoor confined group (ambulatory grade 4 to 6). The ASA score was divided into two groups with the lower risk group (ASA grade 1 and 2) including patients with healthy or mild systemic disease, and the higher risk group (ASA grade 3 to 5) including patients with severe non-incapacitating systemic disease and severe incapacitating systemic disease with a constant threat to life^24^. LOS within the range of 6.15 days to 14.07 days was regarded as normal range of stay, where the mean length of stay of hip fracture patients in Hong Kong is also within this range^25^. Patients before 6 days were regarded as early-discharge and after 14 days as delayed-discharge. Based on this standard, patients were divided into 3 groups: LOS 1 (≤ 6 days); LOS 2 (6 to 14 days); LOS 3 (> 14 days). Delay to surgery was defined as > 48 h from the time of presentation to the time of operation^26^. Complications recorded were presence of urinary tract infection, chest infection, pressure sore, delirium, deep vein thrombosis, wound infection, anemia. After acute hospital stay, patients were transferred to our rehabilitation unit temporarily if not fit for home yet for further arrangements of discharge and rehabilitation. The final destination was recorded as private home and OAH (old age home) groups.

All data were managed using Research Electronic Data Capture (REDCap; Vanderbilt University)^27^. Female gender, pre-fracture mobility indoor confined, low risk ASA, non-delayed surgery, discharge to private home, absence of complications and LOS 2 were set as reference groups. Logistic regression models looking for the prognostic factors affecting the deterioration in mobility were carried out. The first model consisted of the following predictors: age, gender, pre-op ASA, pre-fracture mobility (6 grades of ambulatory categories), delay of surgery (more than 48 h of delay), surgical procedures (surgical approaches or no operation performed as anesthetically unfit), length of hospital stay, discharge destination from hospital (home or OAH), and complications (Yes or No). The second model expanded the complications by types, and the third model included fracture types (Table 1). All statistical analyses, with logistic regression, were performed by using IBM SPSS Statistics for Windows, Version 26.0 (Armonk, NY: IBM Corp). A p-value ≤ 0.05 was considered statistically significant.

**Table 1:** Logistic regression models of prognostic factors affecting the deterioration in mobility.

Model	Predictors	r^2^	B	SE	Wald	Exp(B)	95% CI	P value
1		0.30						
	Age		0.03	0.01	21.17	1.03	1.02, 1.05	< 0.01
	Gender		0.45	0.11	16.76	1.57	1.27, 1.95	< 0.01
	Pre-op ASA		NS					
	Pre-fracture mobility		NS					
	Delay of surgery		NS					
	Surgical procedure							
	No operation performed		1.11	0.56	3.91	3.05	1.01, 9.19	0.05
	Length of stay		NS					
	Discharge destination from hospital							
	OAH		1.52	0.45	11.40	4.59	1.90, 11.11	< 0.01
	Complications^a^		NS					
2		0.30						
	Age		0.03	0.01	20.98	1.03	1.02, 1.05	< 0.01
	Gender		0.46	0.11	16.92	1.58	1.27, 1.96	< 0.01
	Pre-op ASA		NS					
	Pre-fracture mobility		NS					
	Delay of surgery		NS					
	Surgical procedure							
	No operation performed		1.11	0.56	3.87	3.03	1.00, 9.13	0.05
	Arthroplasty—unipolar hemi (uncemented)		0.96	0.49	3.87	2.60	1.00, 6.76	0.05
	Length of stay		NS					
	Discharge destination from hospital							
	OAH		1.45	0.46	9.91	4.26	1.73, 10.49	< 0.01
	Complications^b^		NS					
3		0.30						
	Age		0.03	0.01	20.87	1.03	1.02, 1.05	< 0.01
	Gender		0.46	0.11	17.05	1.58	1.27, 1.97	< 0.01
	Pre-op ASA		NS					
	Pre-fracture mobility		NS					
	Delay of surgery		NS					
	Surgical procedure							
	No operation performed		NS					
	Arthroplasty—unipolar hemi (uncemented)		0.97	0.49	3.97	2.64	1.02, 6.84	0.05
	Length of stay		NS					
	Discharge destination from hospital							
	OAH		1.54	0.46	11.45	4.67	1.91, 11.39	< 0.01
	Complications^b^		NS					
	Fracture type^c^		NS					

Dependent variable: Deterioration in mobility (Ref: No): Key: ASA: American Society of Anesthesiologists, OAH: Old Age Home, NS: Not significant.

Predictors: In Model 1: Gender: Male (Ref: Female). Pre-op ASA: Higher risk (Ref: Lower risk). Pre-fracture mobility: Grade 1 Fully ambulatory, Grade 2 Ambulation with 1 aid, Grade 3 Ambulation with frame, Grade 4 Indoor confined, Grade 5 Standing only (Ref: Non-ambulatory). Delay of surgery: Yes (> 48 h) (Ref: No). Operation: Arthroplasty—bipolar hemi (cemented), Arthroplasty—bipolar hemi (uncemented), Arthroplasty—total hip replacement (THR) (cemented), Arthroplasty—THR (uncemented), Arthroplasty—unipolar hemi (cemented), Arthroplasty—unipolar hemi (uncemented), Excisional arthroplasty, Internal fixation—cannulated screws, Internal fixation—compression hip screw, Internal fixation—IM fixation (Ref: No operation performed). Length of stay: > 14 days, 7–14 days (Ref: < 7 days). Discharge destination from hospital: Home, OAH (Ref: Others). ^a^Complications: Yes (Ref: No).

In Model 2: ^b^Post-op complications: Urinary tract infection: Yes (Ref: No). Chest infection: Yes (Ref: No). Pressure sore: Yes (Ref: No). Delirium: Yes (Ref: No). Deep vein thrombosis: Yes (Ref: No). Wound infection: Yes (Ref: No). Anemia: Yes (Ref: No).

In Model 3: ^c^Fracture type: Neck of femur fracture: Yes (Ref: No). Intertrochanteric fracture: Yes (Ref: No). Subtrochanteric fracture: Yes (Ref: No).

The Ethical approvals of this study were obtained from the Research Ethics Committee (REC) of all six hospitals (reference numbers: KW/EX-13-094(65-06(CMC)) at the Caritas Medical Centre, KW/EX-13-095(65-06(PMH)) at the Princess Margaret Hospital, CT-209/2013 at the Prince of Wales Hospital, KC/KE-13-0106/ER-2 at the Queen Elizabeth Hospital, UW-13-379 at the Queen Mary Hospital, and NTWC/CREC/1170/13 at the Tuen Mun Hospital). This study protocol complied with the Declaration of Helsinki. Informed consent was exempted by the Research Ethics Committee (REC) because of its retrospective study design to review the existing data.

## Results

There were 2698 patients recorded in the system during the study period. All patients underwent hip fracture surgery unless medically unfit for operation. 412 patients were excluded due to missing data (126 without discharge destination, 369 without mobility status, 36 without ASA score and 7 without surgery status in 48 h; 126 had of these patients had multiple missing data). Of the 2286 patients enrolled for final analysis, 846 patients (37.0% of patients) regained their ambulatory ability to the level before fracture. Patient distribution, statistical analysis and clinical characteristics for the two groups are listed in Table 2. There were 1070 neck of femur fractures, 1130 intertrochanteric fractures and 86 subtrochanteric fractures. A total of 174 cannulated hip screws, 458 compression hip screws, 759 cephalomedullary device, 2 cemented total hip replacements, 1 cementless total hip replacement, 10 cemented bipolar arthroplasty, 17 cementless bipolar arthroplasty, 89 cemented unipolar arthroplasty, 680 cementless unipolar surgeries were performed. 93 patients were not operated due to medical conditions that deemed anesthetically unfit.

**Table 2:** Clinical characteristics and statistical analysis results of different groups of patients stratified by mobility category.

Variables	Mobility regained	Mobility deteriorated	OR	95% CI	p-value
Number of patients	846 (37.0%)	1440 (63.0%)	–	–	–
**Gender**					
Male	287 (41.8%)	400 (58.2%)	1.195	1.070–1.335	0.002
Female*	559 (35.0%)	1040 (65.0%)	–	–	-
**Age (years old)**					< 0.001
Mean ± SD	82 ± 8.3	83 ± 7.4			
**Pre-fracture mobility**					
Outdoor unassisted	178(23.3%)	587(76.7%)	2.556	2.101–3.111	< 0.001
Outdoor with aids	344(32.2%)	725(67.8%)	1.480	1.246–1.757	< 0.001
Indoor confined*	324 (71.7%)	128 (28.3%)	–	–	–
**ASA**					
Lower risk group*	323 (34.5%)	614 (65.5%)	–	–	–
Higher risk group	523 (38.8%)	826 (61.2%)	0.831	0.698–0.988	0.038
**Delayed surgery**					
Yes	322(37.0%)	549(63.0%)	1.003	0.842–1.194	1.000
No*	524(37.0%)	891(63.0%)	–	–	–
**Destination after discharge**					
Home*	509(40.2%)	757(59.8%)	–	–	–
OAH	337(33.0%)	683(67.0%)	1.363	1.147–1.619	< 0.001
**Wound infection**					
Yes	19(31.7%)	41(68.3%)	1.276	0.735–2.212	0.419
No*	827(37.2%)	1399(62.8%)	–	–	–
**Urinary tract infection**					
Yes	57(33.5%)	113(66.5%)	1.179	0.847–1.640	0.364
No*	789(37.3%)	1327(62.7%)	–	–	–
**Pressure sore**					
Yes	36(32.4%)	75(67.6%)	1.236	0.823–1.857	0.316
No*	810(37.2%)	1365(62.8%)	–	–	–
**Chest infection**					
Yes	16(36.4)	28(63.6)	1.029	0.553–1.913	1.000
No*	830(37.0)	1412(63.0)	–	–	–
**Length of stay**					
≤ 6 days	191(37.2%)	313(62.8%)	0.988	0.805–1.214	0.916
6–14 days*	447(37.0%)	760(63.0%)	–	–	–
> 14 days	208(36.2%)	367(63.8%)	1.049	0.862 – 1.277	0.653

*Reference group.

Pre-fracture mobility is the single most significant factor on ambulatory status after surgery. Only 23.3% patients with pre-fracture outdoor unassisted ambulatory status and 32.2% patients with pre-fracture outdoor with aids ambulatory status regained the initial level of mobility at one-year after surgery. Compared to patients with indoor confined mobility, patients with outdoor unassisted and outdoor with aids ambulatory function before fracture had 3- and 1.5-times increased risk of mobility deterioration, respectively (Odds Ratio (OR) = 2.556 and 1.480, 95% Confidence Interval (CI) 2.101–3.111 and 1.246–1.757, both p < 0.001). There is a significant decline in mobility more commonly occurring in patients with good premorbid function.

The place where patients live after discharge is the second most important risk factor affecting ambulatory status after surgery. There were 384 new institutionalized patients to OAH and 524 that were already living at an OAH. Patients living in old age homes had almost 1.4 times increased risk of deterioration when compared to those that lived in their homes (OR = 1.363, 95% CI 1.147–1.619, p < 0.001). The risk also increased for every 10 years of age (OR = 1.831, 95% CI 1.607–2.086, p < 0.001). Further analysis on the relationship between ambulation deterioration and residency also showed that significantly more patients that were newly institutionalized had ambulation deterioration compared to those that had always lived at OAH (OR = 1.19, 95% CI 1.11–1.28, p < 0.001) (Table 3). Patients in the higher risk ASA group shows a decreased risk of ambulation deterioration compared to those in lower risk ASA group (OR = 0.831, 95% CI 0.698–0.988, p = 0.038). Furthermore, patients who suffered from complications did not increased risk of mobility decline at one-year post-surgery. Delayed surgery over 48 h, delayed discharge (> 14 days), early discharge (less than 6 days), and length of stay also did not increased risk of mobility decline. Male patients performed worse in terms of their mobility function after surgery compared to female patients (OR = 1.195, 95% CI 1.070–1.335, p = 0.002).

**Table 3:** Relationship between ambulation deterioration and the residency after geriatric hip fracture after hip fracture.

Residency after hip fracture	Ambulation deterioration		P value
	Yes	No	
Home to OAH	323 (84.1) (46.6)	61 (15.9) (28.4)	< 0.01
OAH to OAH	370 (70.6) (53.4)	154 (29.4) (71.6)	

Odds ratio = 1.19 (95% CI: 1.11, 1.28).

Results from logistic regression analyses showed significant relationships between deterioration in mobility with advanced age (OR = 1.03 (1.02, 1.05), p < 0.01), male (1.57 (1.27, 1.95), p < 0.01), no operation performed (3.05 (1.01, 9.19), p = 0.05), and living at OAH after hospital discharge (OR = 4.59 (1.90, 11.11), p < 0.01). In the second model which expanded the complications, cementless unipolar hemiarthroplasty showed statistical significance (OR = 2.60 (1.00, 6.76), p = 0.05). In the third model, with the addition of fracture types, statistical significance remained for cementless unipolar hemiarthroplasty (OR = 2.64 (1.02, 6.84), p = 0.05). All three models R^2^ values were 30% (Table 1).

There were 13.7% of patients in outdoor unassisted group (ambulatory grade 1) at pre-op scored the same after 1 year of surgery. Patients with pre-op grade 1 and 2 scored most at grade 2 (pre-op grade 1 = 36.8%, pre-op grade 2 = 20.4%) and grade 4 (pre-op grade 1 = 25.6%, pre-op grade 2 = 39.2%) after 1 year of surgery. Patients with pre-op grade 3 and 4 scored most at grade 4 (pre-op grade 3 = 41.8%, pre-op grade 4 = 42.8%) and grade 6 (pre-op grade 3 = 30.9%, pre-op grade 4 = 34.9%) after 1 year of surgery. All patients scored 5 at pre-op were grade 6 after 1 year of surgery. Regarding, the discharge destinations from orthopaedic ward, over 70% of patients with pre-op mobility grade 1 to 4 were discharged to rehabilitation wards. Grade 5 and 6 patients were discharged to OAH.

## Discussion

Several clinically important prognostic factors were assessed for the risk in ambulation deterioration in patients 1-year after a geriatric hip fracture. Results show that premorbid good function, discharging to old age homes especially newly institutionalized patients, increased age, lower ASA score (≤ 2), and male patients, correlate with mobility deterioration at one-year post-surgery. Our logistic regression showed that patients with advanced age, male, no operation performed, and living at OAH after hospital discharge especially newly institutionalized patients had higher likelihood of ambulation deterioration. Specific rehabilitation and exercise regimes may be required to improve the clinical outcomes of patients with poor prognostic risk factors. Further analysis of our logistic regression with specific complications showed cementless unipolar hemiarthroplasty surgeries having more likelihood of ambulation deterioration. This concurs with other studies that have stated the higher risk of revision for cementless fixation, which is largely due to implant subsidence with poor bone quality elderly patients^28^. With the emergence of Fracture Liaison Services (FLS)^1,5,29,30^ as the standard of care, coordinators need to identify these patients, especially those at-risk, promptly for physiotherapy to maximize functional outcome. In fact, the International Osteoporosis Foundation recommendations for FLS aims to treat the bone as well as the rehabilitation of patients for best clinical outcomes^30^.

Previous studies identified delayed surgery over 48 h after admission as a risk factor for ambulation recovery after surgery^31^. In our study, delayed surgery showed no effect on mobility recovery. This finding is also supported by Lee et al. in that no significant relationship was observed between surgical timing and functional recovery^16^. More studies and meta-analyses are required to identify whether this is a risk for poor recovery. However, delayed surgery from the hospital can be surrogate parameters for complications and comorbidities. In fact, it is well documented that these can lead to increased mortality rates as well as nosocomial infections^32^. For the Norwegian Hip Fracture Registry, an observational study of 73,557 patients showed that patients operated with a delay exceeding 48 h had increased 3-day mortality, and 1-year mortality^33^. A previous study by Ryan et al., analyzed a total of 2,121,215 patients with hip fractures between 2000 and 2009, and also showed that surgical delay contributed to patient morbidity and mortality^34^. Similarly, a meta-analysis of 28 prospective studies also showed that early hip surgery within 48 h was associated with lower mortality risk and fewer perioperative complications^26^. It is important to note that even with patients with a high ASA or multiple comorbidities, it may not be possible to fully optimize these before surgery. In fact, it has been shown that unnecessary pre-operative cardiology consultations can delay time to surgery of hip fractures^35^. Therefore, following guidelines for optimal management of these patients would aid in decreasing mortality.

Pre-fracture mobility status is the most important predictor of mobility status recovery after surgery. Compared to patients with indoor confined mobility, patients with outdoor unassisted and outdoor with aids ambulatory function before fracture had 3- and 1.5-times increased risk of mobility deterioration, respectively. Concurring with our results, similar results were also reported by Pioli et al., as patients with good premorbid function recovered more slowly than patients with pre-fracture walking limitations^18^. Understandably, patients that have good premorbid function (e.g. walks unaided independently), would have more difficulty to regain this level of mobility as compared to a patient with a poor premorbid function. Pajulammi et al. also reported that pre-fracture mobility level of assisted outdoors and indoor walkers were associated with a smaller decrease in mobility level at one-year follow-up when compared to outdoors unassisted patients^36^. Based on these findings, we can be confident that patients with good pre-fracture ambulatory status deteriorate easily after a geriatric hip fracture. Therefore, we would recommend prompt identification and intensive rehabilitation to patients with good pre-fracture function.

In this study, destination after discharge significantly affected patient ambulatory recovery after one-year of fracture. Patients living in old age homes were 3 times more likely to deteriorate in ambulatory status compared to those that lived at home. Previous studies have shown that not discharging to home was an independent risk factor for failure to regain the pre-fracture level of mobility^36^. Furthermore, a decreased length of stay is associated with reduced rates of mortality. In fact, an increased hospital stay for a hip fracture patient is associated with 32% increased odds of death 30 days after discharge^37^. It has been postulated that patients who can live at home have better health conditions, a higher social-economic class and receive more family support, thereby having improved recovery. Furthermore, it can be assumed that patients who live in old age homes generally have higher frailty and possibly other factors detrimental to recovery of ambulation. On the other hand, institutionalization has variable outcomes due to number of caring staff, socioeconomic status and old age home conditions. A previous systematic review showed that the use of physiotherapy in old age nursing homes varies widely, ranging from 10 to 67%^38^. This would potentially lead to poor function and decline of mobility. Therefore, it has also been recommended that there is a need for developing benchmarks for this to maintain quality standards for patients^38^. Given the aging population, a global call to action is required to not only address the prevention of imminent risk of secondary fractures^39^, but also the functional outcome of patients. The authors would recommend education and care support for patients living at old-aged home by healthcare providers.

Longer hospital stay is strongly correlated with poor mobility outcome in hip fracture patients^40,41^. These have also been reported to cause higher hospital costs as well. Previous studies had reported a higher ASA score as predictors of worse functional recovery, but its long-term 1-year impact on ambulation recovery has not been well reported^42,43^. On the other hand, our findings indicate that a lower ASA score affects the long-term recovery of ambulation in geriatric hip fracture patients. It is likely that patients having better health condition with lower ASA scores tend to have better premorbid function and therefore deteriorates more in terms of mobility after a hip fracture. We also found that male patients were of higher risk of deterioration for mobility. This phenomenon may explain the cause of increased mortality for male patients compared to female patients after a hip fracture in several studies^44,45^. Deterioration has been proven to lead to increased healthcare costs and causes a huge socioeconomic burden^46^.

Assessment of ambulatory status of patients after a hip fracture varies between studies. Several studies used subjective measures by self-rated scores^17^, whilst other studies had objective assessments with the use of ambulatory assistive devices^16^. Recent studies have also assessed ambulation based on the ability of patients being able to participate in outdoor activities, such as outdoor unassisted group, outdoor with aids group and indoor confined group. The authors also use this method as we believe it best reflects the actual activity of a hip fracture patient^18,36,47^. This can also reflect impact of geriatric hip fracture on both the patient and caregiver.

The strengths of this study are that it assesses on many risk factors, and most existing studies only concentrate on fewer and have less patients, to the hundreds only^16,19,21^. This study has included 2286 patients from six acute hospitals involving 90% of all hip fractures in Hong Kong, therefore providing comprehensive and reliable statistical analysis^25^. Furthermore, there is currently a lack of studies focusing on the risk factors in the Chinese population. More importantly, recent studies have predicted that the number of hip fractures in Asia will increase from 1,124,060 in 2018 to 2,563,488 in 2050, with a 2.28-fold increase^22^. 50% of these hip fractures are estimated to occur in China, and therefore it is crucial to for studies to identify these specific risk factors for improvement of ambulatory status to improve patient care and quality of life. We present the first large scale study in a Chinese population, which will potentially address a significant number of future patients. The risk factors identified can also be of important use for clinicians to take caution on.

However, there are several limitations due to the retrospective design, which can weaken the quality of the study. There were different types of hip fractures and therefore it is not uniform, and also there were missing data that required exclusion. Pre-fracture cognitive function and mental status were also not routinely performed for our patients and therefore were not analyzed. It has been reported that these factors have a significant effect on ambulation recovery after hip fracture^41,48^. Our data set did not record all the medications of patients. Side effects from drugs is a common problem in geriatric patients that can affect functional recovery. These can also be confounding factors.

In conclusion, this study identified that better premorbid good function, discharge to old age homes especially newly institutionalized patients, increased age, lower ASA score, and male patients, correlate with mobility deterioration at one-year post-surgery. With the aging population and development of FLS, prompt identification of at-risk patients should be performed for prevention of deterioration.
